# When genomics meets geopolitics

**DOI:** 10.1057/s41599-026-07847-z

**Published:** 2026-06-08

**Authors:** Weijia Ding, Simcha Jong

**Affiliations:** Global Business School for Health, University College London, London, United Kingdom

**Keywords:** Politics and international relations, Scientific community

## Abstract

International genomic collaboration is being reorganised under growing pressure from data sovereignty, geopolitical scrutiny, and demands for fairer benefit-sharing. This Comment uses BGI Group, formerly known as the Beijing Genomics Institute, as a geopolitically salient case to examine how genomic collaboration is being renegotiated around data residency, local sequencing capacity, and jurisdictional control. It does not treat BGI as a representative case, nor does it argue that genomic science has moved from openness to national closure. Rather, BGI helps make visible a broader governance challenge: how to sustain international collaboration when sensitive genomic data is increasingly tied to national laws, regional infrastructures, and expectations of local benefit. This comment argues for sovereignty-compatible interoperability: governance arrangements that allow genomic data to remain under legitimate jurisdictional control while preserving shared standards, reciprocal access, benefit-sharing, and accountable cross-border cooperation. The policy challenge is therefore not to restore an imagined model of frictionless openness, but to build trust infrastructures that combine local legitimacy with international scientific connectivity.

## Introduction

### Genomics is becoming geopolitics

This does not mean that international research has moved neatly from an earlier period of frictionless openness into a new era of national fragmentation. Rather, a renegotiation is unfolding in the governance of global scientific collaboration. Social science studies of genomics and biobanking have long shown that collaboration is shaped not only by ideals of openness, but also by institutional interests, ownership claims over samples and data, public trust, benefit-sharing disputes, and forms of “data hugging” (Tupasela, 2021). These long-standing tensions are especially visible in the life sciences, where genomic data is simultaneously treated as a public good, a biomedical resource, a commercial asset, and an object of national or community concern. What is changing, therefore, is not the sudden arrival of sovereignty in genomics, but the growing visibility and institutional significance of questions around data residency, infrastructural capacity, legal jurisdiction, and trust-building in cross-border scientific collaboration. The argument is therefore not that genomic research has uniformly shifted from openness to closure. Rather, it concerns the changing governance conditions, strategic framings, and institutional justifications through which genomic collaboration is organized and legitimated.

This Comment uses BGI Group as a geopolitically salient case to examine how data sovereignty and geopolitical scrutiny are changing the terms on which genomic collaboration is organized. Formerly known as the Beijing Genomics Institute, BGI Group is a leading life sciences organization with a long-standing role in large-scale genome sequencing and international collaboration, making it a useful case through which to examine these pressures. Through its participation in global genomics initiatives and its more recent regional partnerships, BGI provides a useful lens through which to examine how collaboration is being renegotiated under sovereignty-conscious conditions. These developments show that genomic infrastructures are not merely technical platforms; they are also sites where trust, jurisdiction, and political accountability are negotiated. Importantly, the tensions visible in the BGI case are not unique to China. The issue is therefore not whether genomics is moving from global to regional science, but how global collaboration can be sustained when data, infrastructure, and benefit-sharing are increasingly tied to jurisdictional control.The policy challenge is therefore not to restore an imagined model of frictionless openness, but to build governance infrastructures that sustain trust in cross-border scientific collaboration while combining local legitimacy with international scientific connectivity.

## Scientific decoupling and the rise of data sovereignty

Concerns about scientific decoupling have become more visible in policy debates, particularly in sensitive domains such as artificial intelligence, quantum technologies, and genomics (OECD, 2025; Royal Society, 2023). These concerns extend beyond restrictions on personnel and funding to include contested technical standards, divergent ethical frameworks, and fragmented data infrastructures. Norms of interoperability and open access continue to matter, but they now coexist more visibly with national security concerns, geopolitical competition, and diverging regulatory regimes. In this context, data has emerged as a sovereign asset (Kabata and Thaldar, 2023; WHO, 2024). Genomic data exemplifies these pressures because it is at once a medical resource, a commercial commodity, and a strategic national asset. As debates over benefit-sharing and community data rights intensify, questions of who controls genomic data, under what legal regime, and to whose benefit have moved to the center of global policy discourse (WHO, 2024; Wang, 2025).

This Comment draws on publicly available policy reports, corporate and institutional materials, and published secondary literature in science and technology studies, genomics governance, and biobanking. It does not present new empirical findings. Claims about BGI’s activities and partnerships are grounded in public sources, while the broader argument is situated in the social science literature on genomics and biobanking. The scope of the argument is deliberately limited in three ways. First, this comment does not provide a comprehensive account of all genomic institutions, nor does it claim to demonstrate a uniform global shift in scientific practice. Second, BGI is used as an analytically salient case rather than as a statistically representative case. Third, the argument concerns changing governance conditions, strategic framings, and institutional justifications of collaboration, rather than a simple empirical claim that everyday genomic science has moved from international openness to national closure.

BGI is not treated here as statistically representative of all genomic institutions. Rather, its scale, institutional context, and exposure to geopolitical scrutiny make it a geopolitically salient and strategically revealing case. Earlier debates around the Icelandic Health Sector Database, UK Biobank, BBMRI-ERIC, Generation Scotland, and biobanking initiatives in Singapore have shown that health data infrastructures have long been entangled with questions of public trust, ownership, access, national value creation, and benefit-sharing (Argudo-Portal and Domènech, 2020; Ballantyne et al., 2022; Milbourn et al., 2024; Tupasela, 2021). Read against this broader literature, BGI offers a useful lens through which to examine how genomic collaboration is being renegotiated around data residency, infrastructural capacity, jurisdictional control, and geopolitical trust.

BGI’s relevance also lies in its long-standing role in large-scale sequencing and international genomics. As the first Chinese institution to participate in the Human Genome Project, BGI symbolically marked China’s entry into big science. By the early 2010s, it had become one of the world’s largest sequencing hubs, supported by the acquisition of 128 Illumina HiSeq 2000 sequencers and engaged in projects spanning human health, agriculture, and biodiversity (Puglisi and Rask, 2024). This history matters because it situates BGI not outside global genomics, but within its earlier infrastructures of openness, scale, and cross-border scientific ambition.

Beyond genomic data at scale, BGI also participated in efforts to shape governance norms in international genomics. As an early and active member of the Global Alliance for Genomics and Health (GA4GH), an international consortium founded in 2013 to develop standards for consent, privacy, data sharing, and interoperability, BGI contributed to discussions on genomic data sharing in Asia and to the Framework for Responsible Sharing of Genomic and Health-Related Data (Global Alliance for Genomics and Health, 2014). Its participation helped bring Asian legal, ethical, and cultural perspectives into debates on consent, access, and benefit-sharing. This history is important because it situates BGI within earlier efforts to build shared standards and cross-border trust, while also illustrating how global genomics initiatives sought to broaden their geographic and normative scope beyond Euro-American settings.

## The reworking of genomic collaboration

Recent BGI-linked collaborations suggest a reconfiguration of international genomics partnership rather than a withdrawal from global science. Collaboration continues, but is increasingly organized around local sequencing capacity, data residency, and alignment with domestic or partner-country health and innovation priorities. China’s National GeneBank (CNGB), which BGI helped to operate, is a useful example of how genomic infrastructures can combine scientific, regulatory, and national policy functions. As a state-authorized facility, CNGB foregrounds domestic data stewardship, regulatory compliance, and capacity-building. The Chinese context matters because BGI operates within an innovation system where genomics is linked to public health, biotechnology development, data governance, and technological self-reliance. This does not make BGI representative of all genomic institutions; rather, it makes the case analytically useful by concentrating tensions between global scientific exchange, state-led innovation, data jurisdiction, and geopolitical scrutiny.

A similar emphasis on local embedding is visible in some BGI-linked regional partnerships. In Thailand, BGI funded the Bangkok Genomics Innovation (BKGI) joint venture to embed sequencing and clinical genomics services within the national health system (BGI Genomics, 2017), suggesting an emphasis on anchoring sequencing services within domestic health priorities rather than relying solely on globally pooled repositories. A similar logic can be seen in BGI-linked collaborations in the Middle East, where national genome initiatives place strong emphasis on in-country sequencing capacity, domestic health innovation priorities, and the development of local genomic infrastructures. Taken together, these examples suggest a more sovereignty-conscious form of collaboration, in which cross-border cooperation continues but is increasingly mediated through local infrastructure, data residency, and alignment with national or regional policy goals. Three overlapping pressures help explain this reorientation: geopolitical pressure, the growing politicization of genomic data, and the pursuit of infrastructural self-reliance.

First, rising geopolitical tensions, especially U.S. scrutiny of Chinese genomics firms, have affected the conditions under which BGI and its partners pursue international collaboration. In 2023, the U.S. government placed several BGI subsidiaries on its Entity List (Puglisi and Rask, 2024), citing national security and data misuse concerns.

These measures restricted access to some U.S. technologies and appear to have increased caution among potential research partners and international consortia (U.S. Department of Commerce, 2023; Reuters, 2023).

Second, genomic data is increasingly discussed not only as a scientific input, but also as a resource tied to value creation, sovereignty, and public accountability. While international genomics programmes have often been praised for lowering sequencing costs and expanding global access, they have also been criticized for models of data extraction and commercialization that reinforce dependency rather than delivering tangible returns to local communities (Mwaka et al., 2022; Mc Cartney et al., 2024; Yakubu et al., 2023). These concerns are particularly acute in the Global South, where civil society actors and scholars argue that genomic collaborations must move beyond one-way data flows to ensure fair benefit-sharing and local participation.

Third, against this background, some BGI-linked partnerships can be understood as alternative modes of international cooperation. One visible feature of these partnerships is infrastructural localization: placing sequencing capacity and data governance arrangements inside partner jurisdictions. The Bangkok Genomics Innovation joint venture in Thailand, for example, localizes sequencing services and embeds data storage within the country’s health system. In the Middle East, BGI-linked collaborations have also been associated with national genome initiatives that emphasize in-country sequencing capacity, data governance, and domestic health innovation priorities. Together, these examples suggest that BGI-linked collaborations increasingly emphasize localized infrastructure, data residency, legal clarity, and mutual capacity-building.

## Reconstructing trust in international scientific collaboration

The BGI case points to a broader challenge in the trust architecture of global science. International initiatives such as the Human Genome Project and GA4GH have promoted norms of openness, interoperability, and multilateral cooperation, even as these norms have long coexisted with national, institutional, and commercial claims over data and samples (Collins et al., 2003; Global Alliance for Genomics and Health, 2014). What is becoming more visible is not the disappearance of openness, but the difficulty of sustaining openness when scientific data and infrastructure are increasingly linked to national security, geopolitical tensions, and demands for fairer governance, especially from the Global South (OECD, 2025; WHO, 2024).

A useful way to frame this challenge is sovereignty-compatible interoperability. This refers to governance arrangements that allow sensitive genomic data to remain under legitimate jurisdictional control while preserving the shared standards, reciprocal access, and accountability mechanisms that enable scientific collaboration. Rather than treating sovereignty and interoperability as opposing principles, this framing asks how global science can remain connected when data access, storage, and use are increasingly mediated by national laws, regional infrastructures, and local expectations of benefit.

This framing points to several areas for governance attention. First, interoperability may need to become more federated and distributed. Genomic collaboration does not always depend on the centralized pooling of samples or raw data across borders. It can also be organized through shared metadata standards, compatible access protocols, trusted research environments, and common ethical review criteria that allow analysis and comparison while keeping sensitive data within legitimate legal jurisdictions.

Second, benefit-sharing becomes central to the legitimacy of collaboration. For countries and communities that provide genomic data, collaboration is more likely to sustain trust when it generates local value through capacity-building, clinical translation, training, infrastructure development, and fair access to downstream applications. This is especially relevant where past models of scientific cooperation have been criticized for extracting samples or data without sufficient local return.

Third, risk governance becomes more credible when it is transparent and proportionate. Concerns about national security, data misuse, and commercial exploitation are unlikely to be resolved through exclusion or infrastructural fragmentation alone. More durable forms of cooperation depend on clear access rules, audit mechanisms, accountability procedures, and explainable criteria for assessing risk. In this sense, research platforms and infrastructures become not only technical systems but also instruments of international engagement and trust-building (European Commission, 2021).

Viewed in this way, BGI-linked collaborations in China, Thailand, and the Middle East need not be read simply as evidence of fragmentation. They illustrate a broader governance problem: how to maintain scientific connectivity when genomic data is increasingly tied to jurisdictional authority, domestic capacity, and geopolitical trust. The central challenge is therefore less about restoring an imagined model of frictionless openness than about designing infrastructures that combine local control with global interoperability.

## Conclusion: rethinking international scientific collaboration

Genomic collaboration is not moving from openness to sovereignty in any simple sense. Rather, geopolitical scrutiny and data sovereignty concerns are making long-standing tensions between data sharing, jurisdictional authority, public legitimacy, benefit-sharing, and accountability more visible and more consequential. BGI makes these tensions visible because of its long-standing role in global genomics, its Chinese institutional context, and its exposure to geopolitical scrutiny. The broader issue is therefore not whether global genomics will remain open or become nationalized, but how scientific collaboration can remain credible and connected when data, infrastructure, and trust are increasingly tied to jurisdictional control (OECD, 2025; UNESCO, 2021).

The future of genomic collaboration is unlikely to be either fully open or fragmented. What may emerge instead is a more conditional form of international cooperation, in which data and sequencing capacity are increasingly embedded within national or regional infrastructures, while collaboration continues through shared standards, reciprocal access, and negotiated forms of trust. Initiatives such as Bangkok Genomics Innovation and national genome programmes in the Middle East illustrate elements of this model: sequencing capacity is increasingly localized, but scientific value still depends on connection across institutions, jurisdictions, and expertise.

For funders, policymakers, and international consortia, the central challenge is to make sovereignty and interoperability work together rather than treating them as competing logics. This involves governance arrangements that preserve legitimate local control while enabling responsible data access, benefit-sharing, transparent risk assessment, and accountability. Fairness is central to this task: collaborations that generate capacity, clinical value, and equitable access to downstream applications are more likely to sustain trust, particularly in Global South contexts where extractive models of data use have been contested (Mc Cartney et al., 2024). The broader lesson is therefore not that global science is retreating into national silos, but that scientific collaboration now requires more deliberate trust-building infrastructures capable of combining local legitimacy with global connectivity.

## Data Availability

No datasets were generated or analysed during the current study.
